# Do Decapod Crustaceans Have Nociceptors for Extreme pH?

**DOI:** 10.1371/journal.pone.0010244

**Published:** 2010-04-20

**Authors:** Sakshi Puri, Zen Faulkes

**Affiliations:** Department of Biology, The University of Texas-Pan American, Edinburg, Texas, United States of America; Yale School of Medicine, United States of America

## Abstract

**Background:**

Nociception is the physiological detection of noxious stimuli. Because of its obvious importance, nociception is expected to be widespread across animal taxa and to trigger robust behaviours reliably. Nociception in invertebrates, such as crustaceans, is poorly studied.

**Methodology/Principal Findings:**

Three decapod crustacean species were tested for nociceptive behaviour: Louisiana red swamp crayfish (*Procambarus clarkii*), white shrimp (*Litopenaeus setiferus*), and grass shrimp (*Palaemonetes* sp.). Applying sodium hydroxide, hydrochloric acid, or benzocaine to the antennae caused no change in behaviour in the three species compared to controls. Animals did not groom the stimulated antenna, and there was no difference in movement of treated individuals and controls. Extracellular recordings of antennal nerves in *P. clarkii* revealed continual spontaneous activity, but no neurons that were reliably excited by the application of concentrated sodium hydroxide or hydrochloric acid.

**Conclusions/Significance:**

Previously reported responses to extreme pH are either not consistently evoked across species or were mischaracterized as nociception. There was no behavioural or physiological evidence that the antennae contained specialized nociceptors that responded to pH.

## Introduction

Nociception is the physiological detection of stimuli that are potentially damaging to tissue [1]–[2]. It is closely correlated, but not identical, to the psychological experience of pain [3], and the relationship between nociception and pain, like any relationship between sensory information and subjective perception, is complex [4]–[5]. Understanding nociception in a particular species has significant implications for the care and welfare of that species, and may create new models for research on human pain. For example, a recent review [6] noted that nobody had yet recorded from mammalian sensory neurons for nociception at the receptor ending because the neurons are too small. Invertebrates may offer more tractable systems for studying nociceptor activation, as they have for other problems in neurobiology.

Most research on nociception has been conducted on mammals [5] and other vertebrates [6]–[7]. Nociception has been documented in multiple invertebrate phyla [6], including annelids [8]–[9], nematodes [10], mollusks [11], and insects [12]–[14]. Nevertheless, the entirety of research on nociception for a given phylum is often represented by very small numbers of species [6]. While it might be expected that nociception is widespread and robust, nociception varies across species. For example, the chemical capsaicin is commonly used as a noxious stimulus in experiments with mammals [15]–[17] and triggers nociceptors in some invertebrates [8], [10], but is not noxious to *Drosophila melanogaster* [12]. Similarly, naked mole rats (*Heterocephalus glaber*) do not respond to inflammation as other mammals do [18]. Additionally, several of the invertebrate species in which nociception has been documented are limited as models for studying the physiology of individual nociceptors, due to the small size of the animals.

Only recently have any studies directly addressed crustacean nociception [19]–[22], the first of which was behavioural evidence of nociception in prawns (*Palaemon elegans*) [20]. The authors applied acids or bases to an individual's antenna, which is a major tactile organ [23] that is used in exploratory behaviour [24]–[25] in decapod crustacea. Grooming was preferentially directed towards the stimulated antenna, and grooming was reduced if benzocaine, a local anaesthetic, was applied to the antenna before the noxious stimulant. Most of these findings are broadly consistent with nociception in the better-studied vertebrates, but some are not. For example, benzocaine alone caused a significant increase in grooming, which is not consistent with it acting as an anaesthetic. Further, Elwood and Appel [19] claim that “connections from nociceptors to learning centres are found in decapods,” but the word “nociceptor” or any variation thereof never appears in the paper they cite in support of the statement [26].

If decapod crustaceans show nociceptive behaviour, a reasonable hypothesis is that this behaviour is mediated by specialized sensory neurons that are specifically tuned to tissue-damaging stimuli, i.e., nociceptors. In other animals, nociceptors are often polymodal, and can be triggered by extreme pH [27], extreme temperatures [10], [28], or chemical agonists (e.g., capsaicin [8], [10]) in addition to mechanical damage [11]. Nociceptors are also prone to sensitization if they are repeatedly stimulated [6].

**Figure 1:**
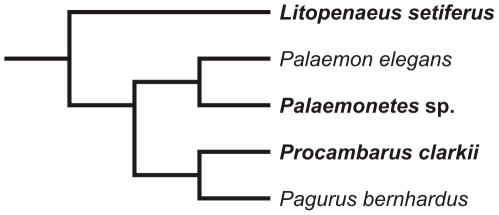
Partial crustacean phylogeny. Phylogenetic relationships of species studied in this paper (shown in bold) and previous studies (*Palaemon elegans*, [20]; *Pagurus bernhardus*, [19], [21]). Phylogeny based on [43]–[45].

Here, we search for evidence of nociception in crustaceans using behavioural and physiological approaches. First, we replicated the behavioural experiments of Barr and colleagues [20] with three other species of decapod crustaceans (Fig. 1): white shrimp (*Litopenaeus setiferus*), grass shrimp (*Palaemonetes* sp.), and Louisiana red swamp crayfish (*Procambarus clarkii*). We hypothesize that nociception should be widespread across many decapod crustacean species, given that nociceptive neurons are widely distributed through the animal kingdom [6], nociception has been suggested to exist in multiple crustacean species [19]–[20], and that these species are likely to encounter similar kinds of noxious stimuli; e.g., mechanical damage from predators, etc.

Second, we recorded the responses of antennal sensory neurons using standard electrophysiological techniques. We hypothesize that if crustacean nociceptors exist, they should have the following physiological properties, based on analogy with nociceptors in other animals [6]. First, crustacean nociceptors should be detectable by extracellular recording; i.e., there is no *a priori* reason to think that nociceptors would be nonspiking or have action potentials so small as to be undetectable. Second, crustacean nociceptors are likely to be tonic excitatory neurons that will show a rapid and sustained increase in the rate of action potentials when exposed to potentially noxious stimuli. Third, crustacean nociceptors are not likely to be more susceptible to damage or death from the noxious stimuli they detect than other sensory neurons in the same region. Given the results of Barr and colleagues [20], we also hypothesize that concentrated acids or bases should be noxious stimuli that will trigger nociceptors, and that nociceptors should be present throughout the antennae.

Earlier versions of this work have been presented in abstract [29].

## Results

### Antenna swabbing

**Figure 2:**
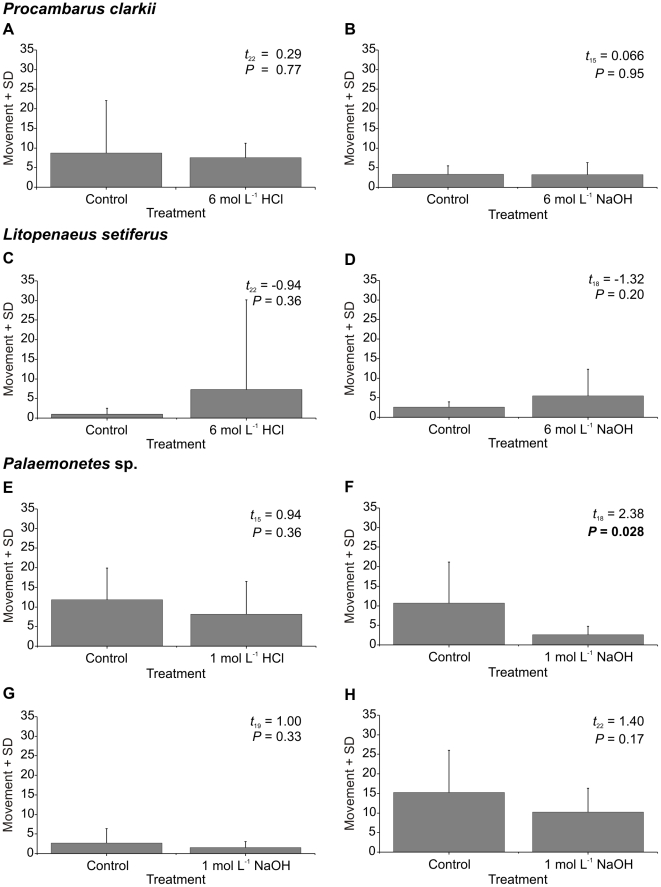
Behaviour following control and noxious stimuli. Movement of animals following application of control or noxious stimuli (HCl or NaOH) to one antenna. (A–B) Crayfish (*P. clarkii*). (C–D) White shrimp (*L. setiferus*). (E–H) Grass shrimp (*Palaemonetes* sp.). Significant probabilities in bold.

Neither *P. clarkii* nor *L. setiferus* showed any significant difference in behaviour following the application of 6 mol L^−1^ NaOH or 6 mol L^−1^ HCl to the antennae compared to the application of controls (Fig. 2a–d). No antennal grooming was seen for either species in either condition, nor were there significant differences in activity (*P. clarkii*: HCl, t_22_ = 0.29, *P = *0.77; NaOH, t_19_ = 1.00, *P = *0.33; *L. setiferus*: HCl, t_18_ = −0.94, *P = *0.36; NaOH, t_18_ = −1.32, *P = *0.20).

*Palaemonetes* sp. (Fig. 2e–h) showed no grooming behaviour following application of either 1 mol L^−1^ HCl or 1 mol L^−1^ NaOH, and no change in activity following application of 1 mol L^−1^ HCl (t_15_ = 0.94, *P = *0.36). An initial experiment (Fig. 2f) showed that application of 1 mol L^−1^ NaOH significantly reduced activity (t_18_ = 2.38, *P = *0.028). Given that this result was not congruent with the other five experiments described above, we tested *Palaemonetes* sp. two additional times (Fig. 2g–h). Neither yielded a significant difference in activity (t_15_ = 0.066, *P = *0.95 and t_22_ = 1.40. *P = *0.17).

**Figure 3:**
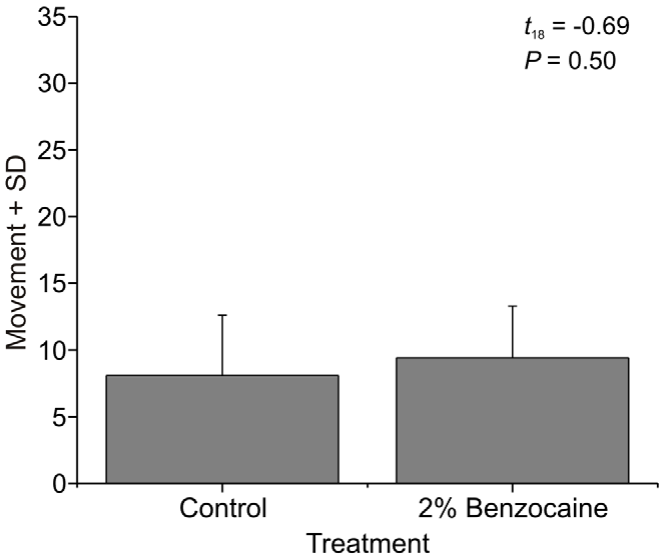
Behaviour following control and benzocaine stimuli. Movement of crayfish (*P. clarkii*) after application of control or 2% benzocaine to one antenna.

Previously, benzocaine alone was found to induce significant directed grooming in *P. elegans* [20]. Applying 2% benzocaine to *P. clarkii* caused no grooming, and no significant difference in activity compared to controls (t_18_ = −0.69, *P = *0.50) (Figure 3).

We did not see the rubbing of antennae against the sides of the tank described by Barr and colleagues [20] in any condition. All touches of the antennae to the tank appeared incidental.

Tailflips occurred too rarely to analyze quantitatively. There were seven instances of tailflipping across all experiments involving all three species. The context in which tailflips occurred suggested that they were responses to handling.

Individuals suffered no long-term effects from the noxious stimuli in any experiment. The treated and control animals' health was not noticeably different in the days after the experiment was conducted.

### Antennal sensory neurons do not respond to extreme pH or benzocaine

We recorded antennal sensory neurons of *P. clarkii* under three conditions: a baseline in which an exposed portion of the antenna was dry; a control condition in which the exposed portion of the antenna was bathed in a putatively innocuous liquid, and; a test condition in which the exposed antenna was bathed in a putatively noxious liquid. If there were nociceptors responding to extremes of pH, we predicted that the noxious stimuli alone would cause a rapid and sustained increase in activity of a neuron, or that a previously silent neuron would begin firing.

**Figure 4:**
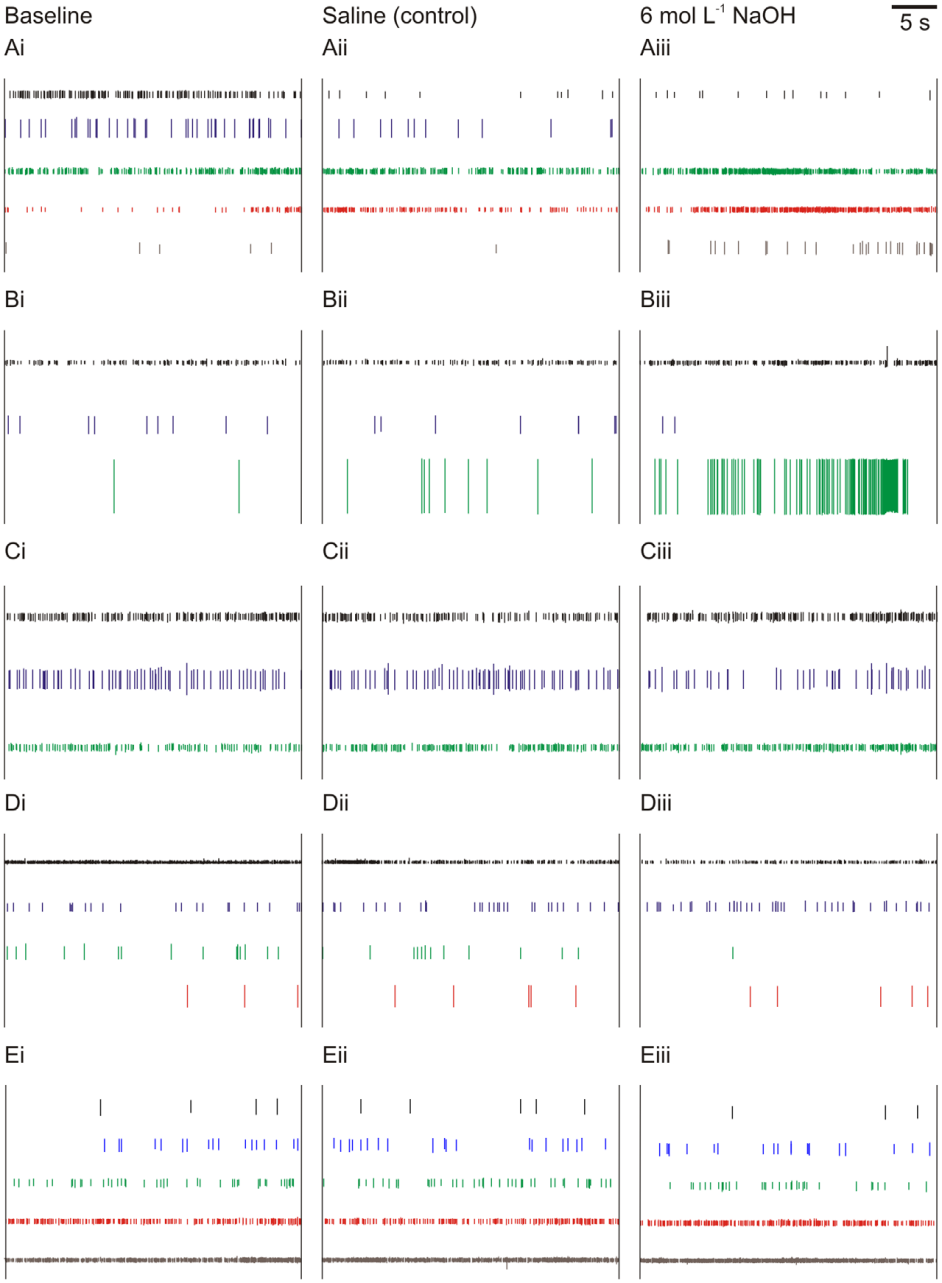
Neural responses to NaOH application. Spikes sorted from extracellular recordings of crayfish (*P. clarkii*) antennal nerves. Each row (A–E) shows one individual; columns (i–iii) show treatment. i  =  baseline; ii  =  application of saline control; iii  =  application of 6 mol L^−1^ NaOH treatment. Heights of different spikes within an individual are proportional to original recording; colours are arbitrary.

**Figure 5:**
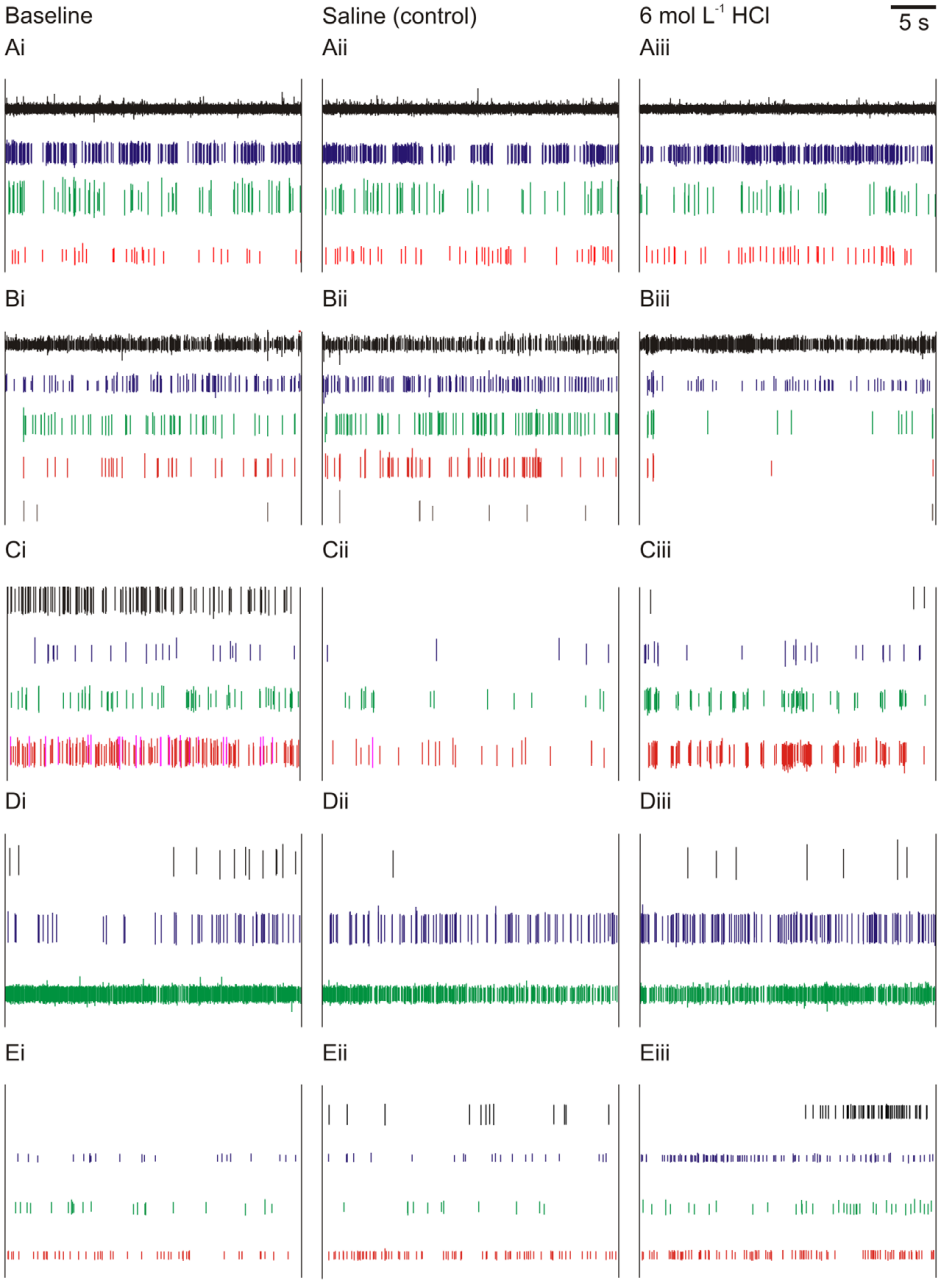
Neural responses to HCl application. Spikes sorted from extracellular recordings of crayfish (*P. clarkii*) antennal nerves. Each row (A–E) shows one individual; columns (i–iii) show treatment. i  =  baseline; ii  =  application of saline control; iii  =  application of 6 mol L^−1^ HCl treatment. Heights of different spikes within an individual are proportional to original recording; colours are arbitrary.

**Figure 6:**
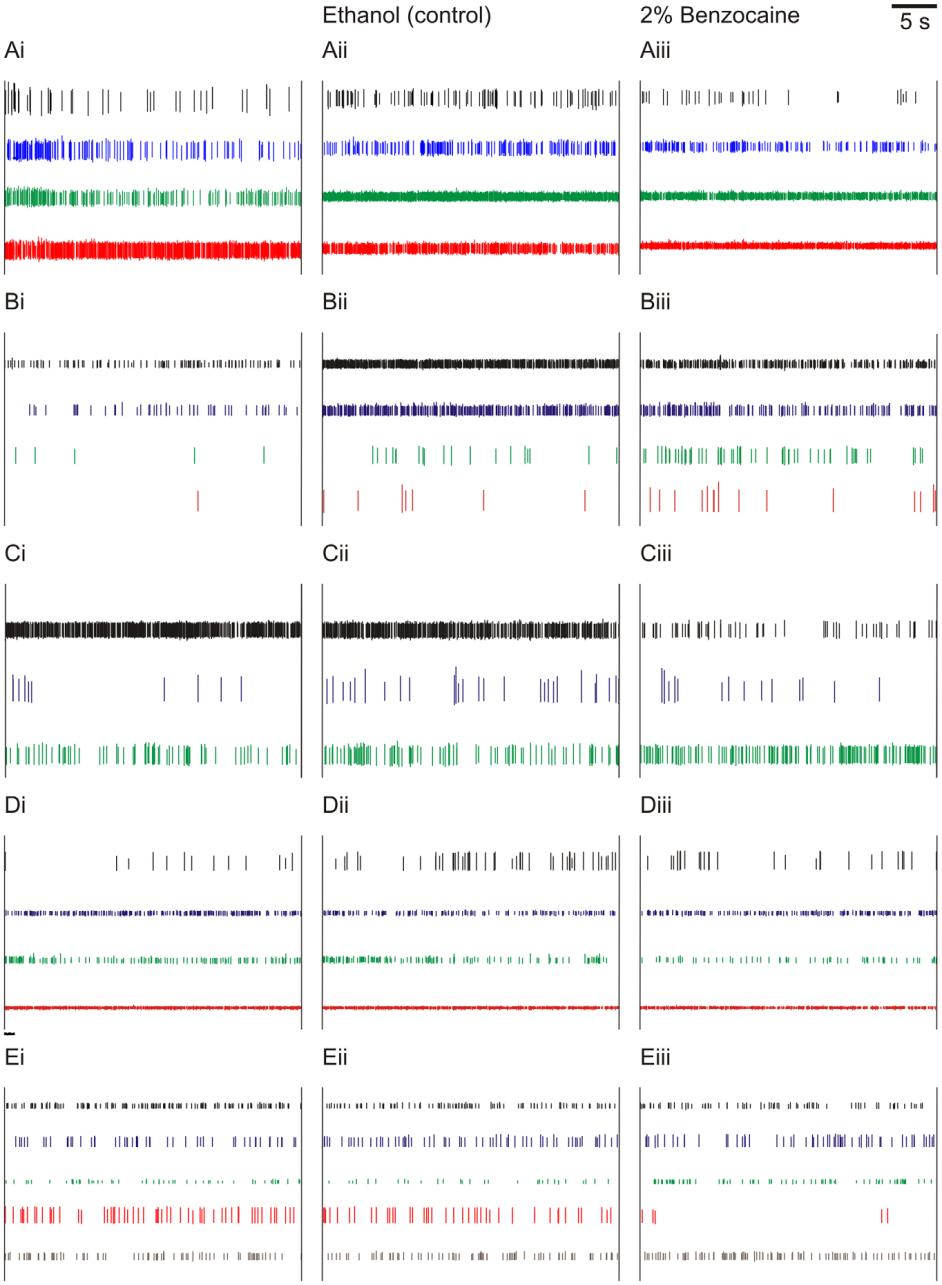
Neural responses to benzocaine application. Spikes sorted from extracellular recordings of crayfish (*P. clarkii*) antennal nerves. Each row (A–E) shows one individual; columns (i–iii) show treatment. i  =  baseline; ii  =  application of ethanol control (necessary due to hydrophobic nature of benzocaine); iii  =  application of 2% benzocaine treatment. Heights of different spikes within an individual are proportional to original recording; colours are arbitrary.

We found no consistent response to 6 mol L^−1^ NaOH (n = 8; five representative recordings shown in Fig. 4), 6 mol L^−1^ HCl (n = 6; five representative recordings shown in Fig. 5), or 2% benzocaine (n = 6; five representative recordings shown in Fig. 6). Although some neurons in some individuals increased their activity in the noxious condition, the variation from individual to individual indicates that these were spontaneous variations in neural activity rather than responses evoked by the noxious stimuli. The neurons remained highly responsive to touch stimuli and water movement throughout the experiments, as has been found in other crayfish species [23], indicating that the recorded neural activity was not merely the random “death throes” of the cells.

### Neural activity is not destroyed by swabbing

**Figure 7:**
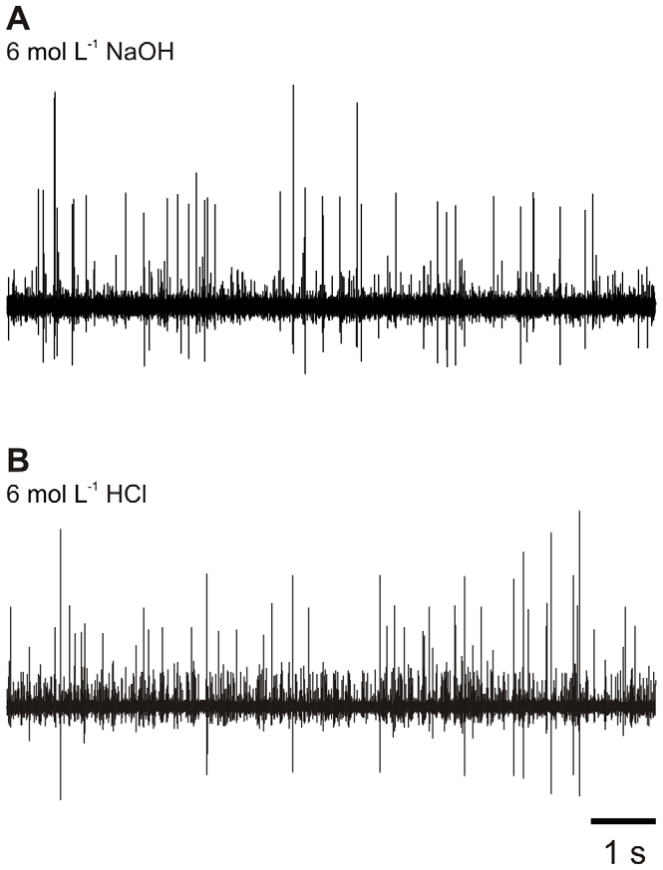
Neural activity following swabbing. Extracellular recordings of crayfish (*P. clarkii*) antennal nerve, taken 10 minutes after swabbing with (A) 6 mol L^−1^ NaOH, (B) 6 mol L^−1^ HCl.

Neuronal activity was recorded in *P. clarkii* antennae after swabbing with either 6 mol L^−1^ HCl or 6 mol L^−1^ NaOH (Fig. 7). Spontaneous activity was present throughout the recording. Clear sensory responses to tactile stimuli or turbulent water flow could be elicited for many tens of minutes, much longer than the ten minute window of observation for the behavioural experiments. Thus, these observations did not support the hypothesis that individuals did not respond to noxious stimuli because many sensory neurons were destroyed by the mechanical act of swabbing.

## Discussion

We found no behavioural or physiological evidence for nociceptors that respond to extreme pH in the antennae. Our results differ from those of Barr and colleagues [20], who reported a significant enhancement of antennal grooming with both benzocaine and weaker acids and bases than used here. We saw essentially no grooming in response to any stimuli.

*Palaemonetes* sp. was the only species that significantly changed behaviour in response to noxious stimuli in one experiment. Although this species is most closely related to the previously studied *P. elegans* [20], we do not consider this result strong evidence supporting nociception in caridean shrimps. First, the reported behaviours are different: *P. elegans* responded to extreme pH by grooming the stimulated region [20], whereas *Palaemonetes* sp. responded to extreme pH by reducing their movement. Second, there is no clear reason why only caridean shrimps should show nociception in these conditions. Neither *P. clarkii* nor *L. setiferus* showed any response to noxious stimuli, even though they were subjected to more intense stimuli than *Palaemonetes* sp., namely a six-fold greater concentration of sodium hydroxide. Third, the effect was not reliably replicated. This, plus the lack of congruence with the results from the other two species and the hydrochloric acid stimuli, suggests that one experiment generated a false positive.

Crustaceans in previous experiments [19]–[21] appear to sense *something* that causes a change in behaviour, and we suggest two possibilities that may reconcile these results. First, we suggest that the behaviour of *P. elegans* [20] may be grooming behaviour rather than nociceptive behaviour. This is consistent with the fact that *P. elegans* grooms in response to benzocaine, an anaesthetic [20], which is not expected if grooming was driven by nociceptive neurons tuned to tissue damage. The differences in the results of Barr and colleagues [20] and this study may be due in part to variation of grooming behaviour in decapods [30]–[33]. Second, we suggest that in experiments using electric shock as noxious stimuli [19], [21], it is possible that animals may be detecting the stimuli using neurons that are not specialized nociceptors [6]. Electric shock has the potential to stimulate any electrically excitable cells (including muscle and other non-neuronal cells, or motor neurons that could convey retrograde action potentials), not just nociceptors. Such difficulties in interpretation underline the need for physiological evidence of nociceptors in crustaceans.

There are various reasons on the face of it to expect crustaceans to have nociception [34], including the widespread distribution of nociception across taxa [6], that crustaceans show avoidance learning [35], and so on. It seems unlikely that nociception would be confined to a few crustacean species, for at least two reasons. First, the sensory capabilities of decapod crustaceans are broadly similar [34], [36]. Second, there is no clear ecological reason why nociception should be present in a patchy pattern across species. We think it unlikely that nociception is found in only a few crustacean species, but there is one clear case of a species with significantly reduced nociception compared to related species. Naked mole rats (*Heterocephalus glaber*) show significantly less nociception than other mammals, and their sensory neurons and neural pathways are quite different than other mammals [18], [37]. Although not predicted in advance, there are several ecological factors that may explain the naked mole rats' unusual features regarding nociception (e.g., carbon dioxide build-up in their subterranean colonies) [6].

Nevertheless, some genetic research points to a possibility that crustaceans may not have nociceptors like those of better studied insects. In *Drosophila melanogaster*, nociception is mediated by a transient receptor potential (TRP) ion channel in the A subfamily, coded by the Pain gene [38]. Orthologs of the *D. melanogaster* Pain gene have been found in five other diverse insect species (silk moths, *Bombyx mori*; flour beetles, *Tribolium castaneum*; honey bees, *Apis mellifera*; parasitoid wasps, *Nasonia vitripennis*; and lice, *Pediculus humanus*). Insects contain four or five TRPA genes, but the crustacean *Daphnia pulex* contains only one [38], indicating that TRPA diversification occurred after the divergence between insects and crustaceans [39]. Thus, it is possible that the nociceptive Pain gene in *D. melanogaster* evolved after the insect-crustacean split, and that the one *D. pulex* TRPA gene is not homologous to the insect Pain gene. Crustacean nociceptors, should they exist, may not be evolutionarily related to those in insects.

We want to make it clear that we are not claiming that crustaceans do not have nociceptors. We are not claiming that crustaceans do not feel pain. Indeed, as we have emphasized, there are many reasons to expect that they could [34], making the results presented here all the more surprising. We are, however, suggesting that the evidence for nociception in crustaceans is still relatively weak, and that the role of nociception in crustacean behaviour may well be neither simple nor straightforward. It is possible that nociceptors may be found in other body areas than the antennae (although one would intuitively expect nociceptors would be found in an animal's major exploratory organ; [24]–[25]). It is also possible that extremes of pH are encountered in aquatic environments so rarely that acids and bases are ecologically irrelevant stimuli that do not evoke a nociceptive response. Nevertheless, nociceptors in other freshwater species, including trout [40]–[41] and leeches [9], respond to external application of acid on the skin, although the current ecological relevance of extreme pH to these species is a matter of some speculation [41]. Other kinds of stimuli, such as mechanical damage, temperature, or selected chemicals, may trigger nociceptive behaviour more readily in decapod crustaceans than extreme pH.

## Materials and Methods

### Animals

*Procambarus clarkii* (Girard, 1852), *Litopenaeus setiferus* (Linnaeus, 1767) and *Palaemonetes* sp. were bought from commercial suppliers, transported to The University of Texas-Pan American and housed in aquaria. *Procambarus clarkii* were housed individually, while *L. setiferus* and *Palaemonetes* sp. were housed communally. Animals of both sexes were used in all experiments.

All experiments were carried out in accordance with federal and state laws and the policies of The University of Texas-Pan American, which exempt research on invertebrates from Institutional Animal Care and Use Committee (IACUC) review.

### Antennal swabbing

Three stimuli used in experiments: sodium hydroxide (NaOH) (which generated the largest effects in prior experiments [20]), hydrochloric acid (HCl) (rather than the acetic acid used in [20]), and benzocaine (C_9_H_11_NO_2_; dissolved in ethanol, as it is not soluble in water). In all experiments, individuals were removed from water and placed on a paper towel. Half the individuals swabbed with a control (water or sea water for NaCl and HCl; ethanol for benzocaine) on the distal half of one second antenna, and half were swabbed the stimulus. Thus, each individual had an antenna that was not swabbed, so that any effects of the mechanical action of swabbing alone could be detected.

Following application of the stimulus, each individual was placed in a small tank for observation. *Litopenaeus setiferus* and *P. clarkii* were observed in tanks 175 mm long ×100 mm wide ×90 mm high. *Palaemonetes* sp. individuals were tested in tanks 200 mm long ×90 mm wide ×150 mm high. These tank sizes are roughly comparable to those used previously [20]. Tanks were filled with ∼50–80 mm of water or sea water (about twice a deep as [20]), except where noted. Behaviour was recorded using a digital video camera (Logitech) to a PC hard drive for 10 minutes, compared to 5 minutes used by [20].

Behaviour was measured in three ways, based on methods in [20]. First, “directed grooming” was measured by contact of other portions of the body (i.e., mouth, legs) with either antenna. Unlike Barr and colleagues [20], we did not include antennae contacting the tank wall in our measure of grooming, as incidental contact seemed highly probable given the small size of the tank and the length of the antennae, particularly in *L. setiferus*. Second, “activity” was measured by counting the number of times the anterior region of the carapace (i.e., eyes) crossed the midline of the tank along its long axis. Third, “tailflips” were recorded. The number of individuals tested in each experiment ranged from 17 to 24. Results were analyzed using unpaired t-tests.

To find a low but effective noxious stimulus, several preliminary trials were made. Preliminary trials on *P. clarkii* and *L. setiferus* with similar acid and base concentrations to those used by [20] generated no detectable responses, so the concentrations were gradually increased during preliminary trials to 6 mol L^−1^, which was the concentration used in all experiments with these two species. *Palaemonetes* sp. individuals were treated with 1 mol L^−1^ NaOH and 1 mol L^−1^ HCl, which is comparable to concentrations used by [20]. *Procambarus clarkii* was the only species tested with 2% benzocaine in ethanol (same concentration as [20]).

No individuals were tested twice. Following their use in these experiments, animals were kept and housed in the lab. Their status was monitored during routine animal care.

Antennae were examined under a dissecting microscope before and after swabbing with water and NaOH to determine if swabbing caused any noticeable alterations in antennal shape, particularly putative sensory hairs.

### Electrophysiology

*Procambarus clarkii* of both sexes were anesthetized by cooling on ice. One second antenna [23] was cut and placed in freshwater crayfish saline composed of (mmol L^−1^) 210 NaCl, 2.5 KCl, 2.5 MgCl_2_, 14 CaCl_2_, and buffered to pH 7.45–7.6 with TRIS [42]. The nerve was exposed by dissection.

We prepared a dish containing a well made from petroleum jelly about 10–20 mm in diameter. The antenna was placed across the top of the well, and was then secured with additional petroleum jelly. Crayfish saline was added to the dish outside the well. The well prevented the liquid being tested (i.e., saline, NaOH, HCl, benzocaine) from interacting with the exposed nerve at the dissected end of the tissue. The nerve tip was placed inside a suction electrode. The recording was allowed to equilibrate for 2 minutes, which established a baseline. A control liquid (crayfish saline for NaOH and HCl experiments; ethanol for benzocaine experiments) was placed in the petroleum jelly well for one minute (control condition). The saline was withdrawn from the well, and the preparation was again allowed to equilibrate for two minutes. Then, the test stimulus (6 mol L^−1^ NaOH, 6 mol L^−1^ HCl, or 2% benzocaine in ethanol) was placed in the well for one minute. The series of treatments (baseline, control liquid, and test liquid, interleaved with equilibration periods) was conducted at least twice for each individual.

Electrical activity was sampled at 20 kHz though a CED 1902 amplifier (Cambridge Electronic Design), HumBug noise filter (Quest Scientific), CED Micro 1401 Mark II analogue-to-digital board (Cambridge Electronic Design), and recorded on a Windows-based PC using Spike 2 version 5.20 software (Cambridge Electronic Design). The spike sorting capabilities of Spike 2 software were used to identify individual neurons on the basis of spike height and shape. For each treatment, we made at least five recordings where we were able to distinguish three spikes or more.

To test whether the mechanical action of swabbing the antennae destroyed the sensory neurons, antenna were dissected from chilled crayfish, as described above. The antenna was swabbed with 6 mol L^−1^ NaOH or 6 mol L^−1^ HCl in the same way as intact animals. The antenna was placed back in saline in a dish, and the nerve tip was placed inside a suction electrode, and recorded as described above.
